# Clinical application of elevated platelet-activating factor acetylhydrolase in patients with hepatitis B

**DOI:** 10.1186/1476-511X-13-105

**Published:** 2014-06-28

**Authors:** Limin Feng, Ying Zhao, Guofang Feng, Yu Chen

**Affiliations:** 1 Department of Laboratory Medicine, The First Affiliated Hospital, College of Medicine, Zhejiang University, Hangzhou 310003, China; 2 Department of Reproductive Endocrinology, Women’s Hospital, School of Medicine, Zhejiang University, Hangzhou 310006, China

**Keywords:** Hepatitis B, Platelet-activating factor acetylhydrolase (PAF-AH), Chronic severe hepatitis B (CSHB)

## Abstract

**Background:**

The aim of this study was to investigate the variation of platelet-activating factor acetylhydrolase (PAF-AH) in patients with various stages of hepatitis B infection and evaluate the association between PAF-AH activity and chronic severe hepatitis B (CSHB) and mortality in patients with hepatitis B.

**Methods:**

Serum PAF-AH activity was measured in 216 patients with hepatitis B and in 152 healthy controls using an automatic biochemical analysis system. Spearman correlation was used to investigate the correlation between PAF-AH activity and other biochemical indicators. The receiver operating characteristic (ROC) curve and multivariable logistic regression analysis were used to evaluate the ability of PAF-AH activity to predict CSHB and mortality in patients with hepatitis B.

**Results:**

The PAF-AH activities in patients with CSHB (1320 ± 481 U/L) were significantly higher than those in healthy controls and in other hepatitis B groups (all *P* < 0.01). In patients with hepatitis B, PAF-AH activity correlated with total bilirubin (r = 0.633), total bile acid (r = 0.559), aspartate aminotransferase (r = 0.332), apolipoprotein B (r = 0.348), high-density lipoprotein cholesterol (r = −0.493), and apolipoprotein AI (r = −0.530). The areas under the ROC curves for the ability of PAF-AH activity to predict CSHB and mortality in patients with hepatitis B were 0.881 (95% confidence interval (CI): 0.824–0.937, *P* < 0.001) and 0.757 (95% CI: 0.677–0.837, *P* < 0.001), respectively. Multivariate logistic regression analysis showed PAF-AH activity to be an independent factor predicting CSHB with an odds ratio of 1.003 (95% CI: 1.002–1.005, *P* < 0.001).

**Conclusion:**

Elevated PAF-AH in patients with hepatitis B was significantly associated with liver damage. Thus, serum PAF-AH could be used as a novel indicator for predicting CSHB and mortality in patients with hepatitis B. Further, PAF-AH activity was an independent factor predicting CSHB.

## Introduction

Platelet-activating factor acetylhydrolase (PAF-AH) is a Ca^2+^-independent catalyst of serine-dependent phospholipid hydrolysis and belongs to the superfamily of phospholipase A2 [1]. Also known as lipoprotein-associated phospholipase A_2_ (Lp-PLA_2_), the plasma PAF-AH is a single 45-kilodalton polypeptide composed of 441 amino acids encoded by the *PLA2G7* gene [2]. Because PAF-AH hydrolyzes platelet-activating factor (PAF) and oxidizes phospholipids with a modified short fatty acyl chain esterified at the sn-2 position [3], PAF-AH plays an important role in human diseases such as severe anaphylaxis [4], rheumatic diseases [5], acute respiratory distress syndrome [6], necrotizing enterocolitis [7], and atherosclerosis [8]. Previous epidemiologic studies demonstrated that increased PAF-AH activity had a prognostic value and was associated with a high risk of future coronary and cerebrovascular events [9,10]. Additionally, Kamisako et al. reported increased PAF-AH activity in patients with hyperbilirubinemic hepatobiliary disease [11]. However, to our knowledge, the role of serum PAF-AH in hepatitis B has not yet been well defined. More importantly, whether serum PAF-AH activities are associated with different disease states of hepatitis B virus (HBV) infection such as acute hepatitis B (AHB), chronic hepatitis B (CHB), and chronic severe hepatitis B (CSHB) remains unknown. Thus, in the present study, we aim to determine the activity of serum PAF-AH in patients with various stages of hepatitis B and to evaluate the association of PAF-AH with different hepatitis B disease groups and with mortality in patients with hepatitis B.

## Methods

### Subjects

A total of 216 hepatitis B patients, including 155 male and 61 female patients aged 13–82 (45.1 ± 13.4) years from the Department of Infectious Disease, The First Affiliated Hospital, School of Medicine, Zhejiang University, China, were enrolled in our prospective study. Of these patients, 23 were diagnosed with acute hepatitis B (AHB), 67 with chronic hepatitis B (CHB), 49 with chronic severe hepatitis B (CSHB), and 77 with liver cirrhosis (LC). All patients were diagnosed according to the criteria of the 2000 Xi’an viral hepatitis management scheme [12]. The standardized diagnosis of AHB, CHB, and CSHB has been previously described in detail [13-15]. The model for end-stage liver disease (MELD) score, calculated from the patient’s serum total bilirubin (TBIL), creatinine level, and international normalized ratio (INR) of prothrombin time, was used to quantify the severity of liver disease [16]. A total of 152 healthy control patients with HBsAg negativity and normal liver and renal function and blood lipid levels at their annual health examination at the healthcare center of The First Affiliated Hospital of Zhejiang University were also recruited. The control group comprised 102 male and 50 female patients aged 17–78 (46.0 ± 12.9) years. Patients with a concurrent infection of hepatitis C virus (HCV), hepatitis D virus, hepatitis G virus, and/or human immunodeficiency virus and any autoimmune liver disease were excluded. There were no statistically significant differences in gender and age distribution between the case and control groups (both *P* > 0.05).

### Ethics statement

This study was approved by the Ethics Committee of the First Affiliated Hospital of College of Medicine at Zhejiang University in China and was performed in accordance with the Helsinki Declaration. All participants provided written informed consent. For participants under 18 years of age, oral informed consent was obtained from the participants, and written informed consent was signed by their parents.

### Specimen collection

All specimens for blood indicators and PAF-AH activity measurements were collected by venipuncture into 5-mL drying Vacuette vacutainers (Greiner Bio-One GmbH, Kremsmunster, Austria) in the morning after a 12 h fast on the second day after admission. The samples were sent to the laboratory, and serum was isolated by centrifugation (10 min, 3000 × *g*) and preserved at −80°C.

### Laboratory techniques

Biochemical indicators of liver function, such as alanine aminotransferase (ALT), aspartate aminotransferase (AST), total bilirubin (TBIL), cholinesterase (ChE), triglyceride (TG), total cholesterol (Tch), low-density lipoprotein cholesterol (LDL-c), high-density lipoprotein cholesterol (HDL-c), apolipoprotein AI (apoAI), apolipoprotein B (apoB), glucose (Glu), and total bile acid (TBA), were assessed on a Hitachi 7600 automatic biochemical analyzer (Hitachi Ltd., Tokyo, Japan) using reagents from Roche Diagnostics (Roche Diagnostics GmbH, Mannheim, Germany) for ALT, AST, TBIL, ChE, TG, and Tch measurements and reagents from SSUF (SSUF Ltd., Shanghai, China) for LDL-c, HDL-c, apoAI, apoB, and TBA measurements. PAF-AH activity was measured by a previously described spectrophotometric assay using the Azwell Auto PAF-AH kit (Azwell Inc., Osaka, Japan). We used a combination of 3-[(3- cholamidopropyl) dimethylammonio]-1-propanesulfonate (CHAPS) and sodium 1-nonane sulfonate to avoid measuring nonspecific esterase activity in our assay [17]. In brief, PAF-AH hydrolyzes the substrate 1-myristoyl-2-(4-nitrophenylsuccinyl) phosphatidylcholine at the sn-2 position, producing 4-nitrophenyl succinate, which is immediately degraded to 4-nitrophenol and subsequently measured spectrophotometrically at 405 nm at room temperature. The enzymatic activity was expressed as IU/L.

### Statistical analysis

Statistical analysis was performed using SPSS software version 13.0 (SPSS Inc., Chicago, IL, USA). Data are presented as mean ± SD, and categorical data as percentages. For continuous variables, the differences between two groups were assessed with the independent samples *t*-test or the Mann–Whitney U test as appropriate. Multiple comparisons were performed by one-way analysis of variance (ANOVA) or Kruskal-Wallis H tests. Categorical variables were analyzed using the chi-square test. Spearman’s rank correlation test was used in correlation analysis. For the univariate and multivariate analyses to identify independent predictors, the measurements for PAF-AH activity, biochemical parameters, and hematological parameters were presented as quartile ranks, with the lowest quartile being used as the reference category. A receiver operating characteristic (ROC) curve was generated and the area under the curve (AUC) was calculated to identify the best PAF-AH activity and/or MELD score for predicting CSHB and mortality in patients with HBV infection. Stepwise regression was performed to determine factors associated with the incidence of CSHB. A multivariable logistic regression analysis was used to evaluate PAF-AH activity and the MELD score as predictors of CSHB by adjusting for gender, LDL-c, and TBIL in the model. Statistical significance was defined at *P* < 0.05.

## Results

### Detection of PAF-AH in patients with various clinical stages of hepatitis B

In this study, 216 patients with hepatitis B and 152 healthy control patients were enrolled as study participants. The clinical characteristics of the patients are listed in Table 1. The levels of ALT, AST, TBIL, CHE, TG, Tch, LDL-c, HDL-c, apoA I, apoB, and TBA were statistically different between each of the AHB, CHB, LC, and CSHB groups and the healthy control group (all *P* < 0.05). The MELD score and mortality were statistically different among the four hepatitis B groups (all *P* < 0.05). The PAF-AH activity was 820 ± 446 U/L in the 216 patients with hepatitis B, significantly higher than that measured in healthy controls, 450 ± 125 U/L (*P* < 0.01). The PAF-AH activities in the AHB, CHB, LC, and CSHB groups were all significantly higher than in control patients (all *P* < 0.05, Table 1). Moreover, the PAF-AH activities in patients with CSHB were significantly higher than those in patients with AHB, CHB, and LC by the Mann–Whitney U test (all *P* < 0.001).

**Table 1:** Clinical characteristics of enrolled participants

**Variable**	**Control (152)**	**AHB (23)**	**CHB (67)**	**LC (77)**	**CSHB (49)**	***P-*** **value**
Female/male	50/102	12/11	18/49	23/54	8/41	0.031
Age (year)	45.1 ± 13.4	40.2 ± 14.9*	39.1 ± 12.2*	50.7 ± 13.4*	44.2 ± 11.8	<0.001
BMI (kg/m^2^)	22.9 ± 2.9	23.1 ± 2.8	23.1 ± 2.9	22.5 ± 3.3	22.9 ± 3.3	0.237
TBIL (μmol/L)	13.1 ± 4.4	126.9 ± 108.5*	58.5 ± 105.2*	76.6 ± 110.8*	423.5 ± 194.6*	<0.001
TBA (μmol/L)	3 ± 2	133 ± 86*	46 ± 69*	65 ± 76*	156 ± 80*	<0.001
ALT (U/L)	19 ± 9	1289 ± 590*	402 ± 478*	82 ± 165*	309 ± 353*	<0.001
AST (U/L)	19 ± 6	516 ± 398*	190 ± 197*	83 ± 74*	242 ± 203*	<0.001
ChE (U/L)	8364 ± 1521	6003 ± 2012*	6887 ± 2235*	3072 ± 1468*	3477 ± 1370*	<0.001
TG (mmol/L)	0.95 ± 0.33	1.95 ± 0.97*	1.45 ± 0.86*	0.95 ± 0.73*	1.42 ± 0.79*	<0.001
Tch (mmol/L)	4.17 ± 0.58	3.46 ± 0.71*	3.67 ± 0.87*	3.16 ± 1.67*	2.62 ± 1.49*	<0.001
HDL-c (mmol/L)	1.33 ± 0.24	0.42 ± 0.41*	0.86 ± 0.45*	0.67 ± 0.45*	0.11 ± 0.15*	<0.001
LDL-c (mmol/L)	2.41 ± 0.47	1.46 ± 0.94*	1.90 ± 0.68*	1.64 ± 0.71*	1.09 ± 0.87*	<0.001
apoAI (g/L)	1.18 ± 0.25	0.61 ± 0.35*	0.95 ± 0.38*	0.64 ± 0.30*	0.21 ± 0.16*	<0.001
apoB (g/L)	0.69 ± 0.15	0.87 ± 0.27*	0.74 ± 0.24*	0.57 ± 0.25*	0.71 ± 0.33*	<0.001
Cr (μmol/L)	71 ± 13	99 ± 156*	74 ± 17	80 ± 69	102 ± 77*	0.111
Glu (mmol/L)	4.76 ± 0.41	4.79 ± 0.81	4.79 ± 0.85	5.45 ± 2.42*	4.86 ± 2.53	0.052
PT (s)	-	12.7 ± 1.48	12.2 ± 2.59	16.3 ± 5.2	23.5 ± 9.6	<0.001^#^
PAF-AH (U/L)	450 ± 125	872 ± 355*	614 ± 315*	666 ± 267*	1320 ± 481*	<0.001
MELD score	-	11.9 ± 5.9	6.9 ± 5.7	10.7 ± 7.0	24.7 ± 9.1	<0.001^#^
Mortality (yes/no)	-	0/23	0/67	7/70	27/22	<0.001^#^

*AHB*: acute hepatitis B; *CHB*, chronic hepatitis B; *LC*, liver cirrhosis; *CSHB*, chronic severe hepatitis B; *Cr*, creatinine; *PT*, prothrombin time.

*P*-value: comparison among these five groups. ^#^comparison among these four groups. *P < 0.05 vs. the control group.

### Correlation analysis between PAF-AH activity and other laboratory indexes

**Figure 1:**
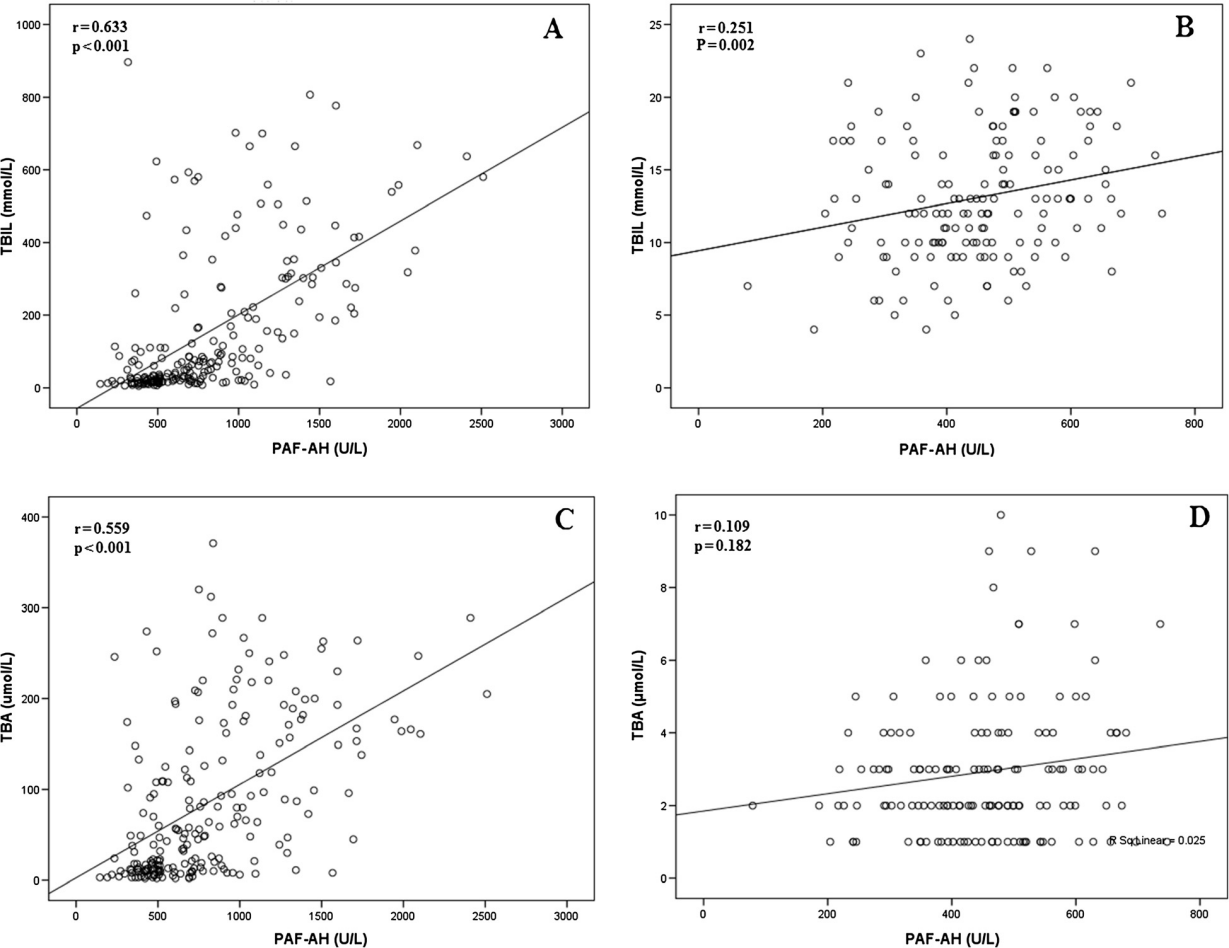
**Correlation analysis between serum PAF-AH and TBIL (A, B) and TBA (C, D) in patients with hepatitis B (A, C) and in healthy controls (B, D).** Serum PAF-AH, TBIL, and TBA were measured by spectrophotometric assays in 152 healthy patients and 216 patients diagnosed with hepatitis B. The Spearman correlation coefficients between these variables were determined as shown on the plots. No significant correlation was detected between PAF-AH activity and TBA in healthy controls, but TBIL levels correlated positively with PAF-AH activities in both healthy controls and hepatitis B–positive patients. TBA also correlated positively with PAF-AH in hepatitis B–positive patients.

Spearman correlation was employed to determine the correlation between PAF-AH activity and other laboratory indexes. We found that in hepatitis B–positive patients, PAF-AH activity correlated positively with TBIL, TBA, ALT, AST, TG, and apoB (Spearman correlation coefficients [r-values] = 0.633, 0.559, 0.176, 0.332, 0.276, and 0.348, respectively; all *P* < 0.05), correlated negatively with ChE, HDL-c, and apoAI (r = −0.273, −0.493, and −0.530, respectively; all *P* < 0.05), and did not correlate with Glu, body mass index (BMI), Tch, or LDL-c levels (all *P* > 0.05). Both TBIL and TBA levels were very significant correlated with serum PAF-AH activity in hepatitis B patients (r-values greater than 0.5, Figure 1). In contrast, in healthy controls, PAF-AH activity correlated positively with TBIL, ALT, ChE, TG, Tch, LDL-c, and apoB (r = 0.251, 0.244, 0.209, 0.200, 0.338, 0.416, and 0.436, respectively; all *P* < 0.05), correlated negatively with HDL-c (r = −0.176, *P* < 0.05), and did not correlate with Glu, BMI, TBA, AST, and apoAI levels (all *P* > 0.05).

### Association of PAF-AH with CSHB and 3-month mortality in HBV-infected patients

To explore the association of PAF-AH with CSHB and with mortality, the 216 patients with hepatitis B were divided into four groups according to their PAF-AH activity percentiles (group 1: PAF-AH >1053 U/L; group 2: 709–1053 U/L; group 3: 477–708 U/L; group 4: <477 U/L). The prevalence of CSHB was calculated by dividing the number of patients with CSHB by the total numbers of patients in each PAF-AH percentile group. The clinical characteristics and differences in measurements for variables among the four groups are listed in Table 2. Patients with the highest values of PAF-AH (group 1) had higher MELD scores, incidence of CSHB, and mortality than patients with the lowest values of PAF-AH (group 4) (20.7 ± 9.9 vs. 7.0 ± 5.5, 35/19 vs. 1/53, and 17/37 vs. 1/53, respectively; all *P* < 0.05).

**Table 2:** **Clinical characteristics of patients with hepatitis B according to serum PAF**-**AH percentiles**

**Variable**	**Group 1**	**Group 2**	**Group 3**	**Group 4**	***P-*** **value**
PAF-AH (U/L)	>1053	709-1053	477-708	< 477	
Female/male	14/40	13/40	25/30	18/36	0.077
Age (year)	44.6 ± 12.4	42.3 ± 12.5	45.1 ± 13.6	45.9 ± 16.1	0.574
BMI (kg/m^2^)	22.8 ± 3.6	22.8 ± 2.5	22.7 ± 2.3	23.0 ± 3.5	0.248
TBIL (μmol/L)	337.4 ± 206.9	139.1 ± 161.9	85.6 ± 148.3	59.1 ± 137.2	<0.001
TBA (μmol/L)	151 ± 79	110 ± 99	51 ± 56	37 ± 59	<0.001
ALT (U/L)	365 ± 456	507 ± 699	333 ± 454	243 ± 374	0.067
AST (U/L)	276 ± 264	223 ± 280	174 ± 201	121 ± 150	<0.001
ChE (U/L)	3766 ± 1905	4400 ± 2631	5000 ± 2716	5470 ± 2436	0.003
TG (mmol/L)	1.68 ± 0.94	1.35 ± 0.90	1.19 ± 0.59	1.04 ± 0.89	<0.001
Tch (mmol/L)	3.11 ± 1.48	3.51 ± 1.76	3.37 ± 1.23	2.94 ± 0.90	0.147
HDL-c (mmol/L)	0.22 ± 0.37	0.54 ± 0.49	0.69 ± 0.43	0.83 ± 0.44	<0.001
LDL-c (mmol/L)	1.28 ± 0.97	1.73 ± 0.91	1.80 ± 0.70	1.53 ± 0.57	0.005
ApoAI (g/L)	0.30 ± 0.27	0.60 ± 0.35	0.79 ± 0.40	0.85 ± 0.37	<0.001
ApoB (g/L)	0.82 ± 0.34	0.73 ± 0.31	0.67 ± 0.21	0.53 ± 0.16	<0.001
Cr (μmol/L)	89 ± 69	87 ± 84	94 ± 104	70 ± 13	0.323
Glu (mmol/L)	4.49 ± 1.84	5.67 ± 2.87	5.16 ± 1.57	4.86 ± 1.97	0.015
PT (s)	20.5 ± 9.4	17.2 ± 7.6	13.8 ± 3.9	13.9 ± 4.3	<0.001
MELD score	20.7 ± 9.9	14.0 ± 9.1	10.1 ± 8.2	7.0 ± 5.5	<0.001
Mortality (yes/no)	17/37	10/43	6/49	1/53	<0.001
CSHB (yes/no)	35/19	8/45	5/50	1/53	<0.001

**Figure 2:**
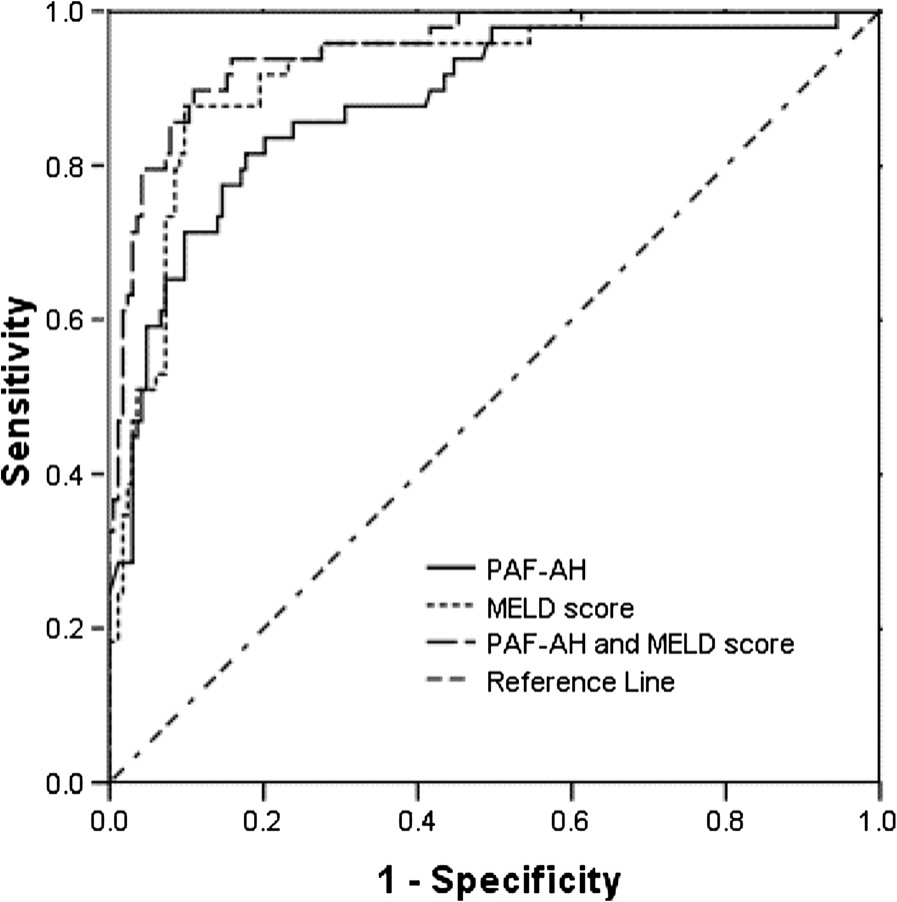
ROC curve analysis for predicting CSHB in hepatitis B–positive patients by serum PAF-AH and the MELD score.

ROC curve analysis was applied to estimate the ability of PAF-AH activity and the MELD score to predict CSHB in patients with hepatitis B (Figure 2, Table 3). The AUCs of PAF-AH activity and the MELD score were 0.881 (95% confidence interval (CI): 0.824–0.937, *P* < 0.001) and 0.921 (95% CI: 0.879–0.962, *P* < 0.001), respectively. When PAF-AH and the MELD score were combined, the AUC was 0.951 (95% CI: 0.919–0.982, *P* < 0.001).

**Table 3:** Cutoff values, sensitivity, and specificity of serum PAF-AH and the MELD score in predicting CSHB and mortality in hepatitis B patients

	**Cutoff values**	**Sensitivity (%)**	**Specificity (%)**
Prediction of CSHB			
PAF-AH (U/L)	910	81.6	82.2
MELD score	16.7	87.8	90.2
PAF-AH + MELD score		89.8	89.0
Prediction of mortality			
PAF-AH (U/L)	799	76.5	66.5
MELD score	22.6	70.5	94.9
PAF-AH + MELD score		70.6	94.9

**Figure 3:**
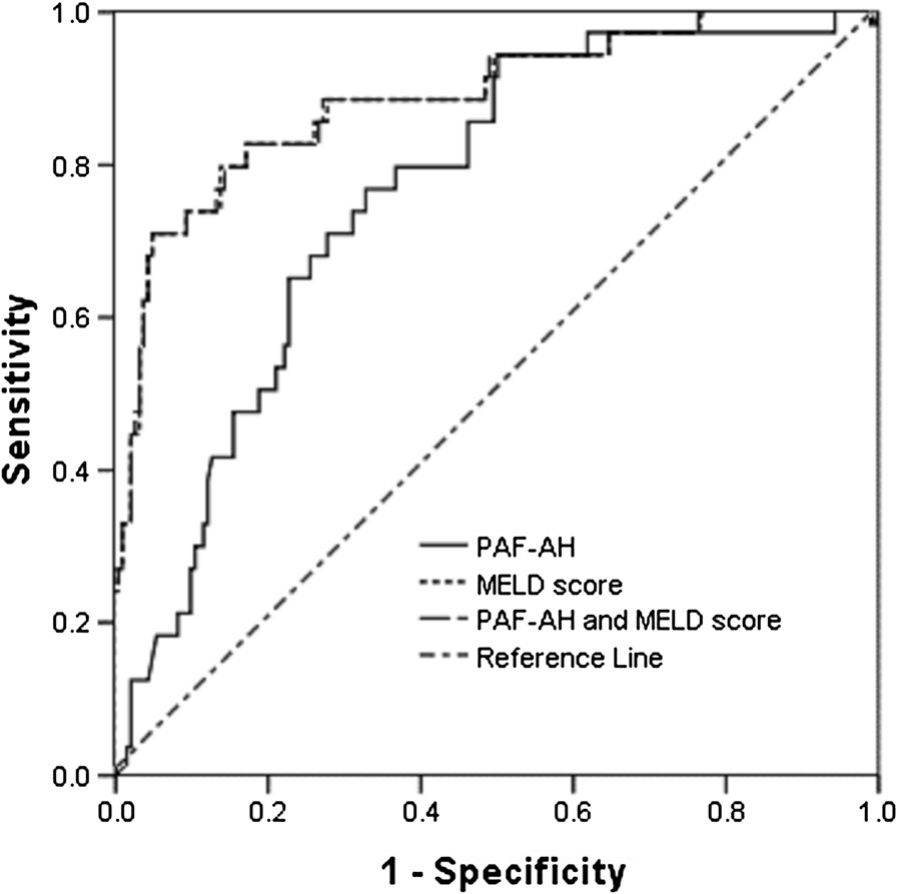
ROC curve analysis for predicting mortality in hepatitis B–positive patients by serum PAF-AH and the MELD score.

Further ROC curve analysis was performed to estimate the ability of PAF-AH activity and the MELD score to predict mortality in patients with hepatitis B (Figure 3, Table 3). The AUCs of PAF-AH activity and the MELD score were 0.757 (95% CI: 0.677–0.837, *P* < 0.001) and 0.883 (95% CI: 0.814–0.952, *P* < 0.001), respectively. When PAF-AH and MELD were combined, the AUC was 0.882 (95% CI: 0.815–0.952, *P* < 0.001).

Gender, LDL-c, TBIL, the MELD score, and PAF-AH were found to be associated with the prevalence of CSHB (*P* = 0.016, 0.038, <0.001, 0.007, and <0.001, respectively) by stepwise regression in which variables were selected from age, gender, PAF-AH, ALT, AST, TBIL, ChE, TG, Tch, LDL-c, HDL-c, apoAI, apoB, and TBA. Further multivariate logistic regression analysis following adjustment for gender, LDL-c, and TBIL showed that PAF-AH activity and the MELD score were independent factors predicting CSHB, with odds ratios of 1.003 (95% CI: 1.002–1.005, *P* < 0.001) for PAF-AH and 1.152 (95% CI: 1.037–1.279, *P* = 0.011) for the MELD score.

## Discussion

PAF is a potent inflammatory lipid mediator that, by binding to a high-affinity G-protein–linked receptor, can be activated to exert diverse actions including stimulating secretion and aggregation of platelets, initiating neutrophil and macrophage chemotaxis, and inducing release of cytokines such as interleukins, tumor necrosis factor, and proteolytic enzymes, and thus may be involved in the development of circulation disorders and inflammation [18]. PAF accumulation has been implicated in pathological processes and diseases including inflammation, endotoxin shock, acute pancreatitis, and cardiovascular disease. PAF is also involved in acute liver damage, cirrhosis, severe hepatitis, and ischemia-reperfusion–induced liver injury [19,20]. As a major mediator of PAF inactivation, PAF-AH plays a crucial role in the regulation of serum PAF levels and in reducing PAF-induced damage [8,21-23]. In experimental models of acetaminophen-induced liver injury in rats, PAF activities increased significantly between 24 and 32 h after acetaminophen administration, along with increases in other biochemical indexes (ALT, AST). The PAF-AH activity peaked between 72 and 96 h after acetaminophen treatment [24], indicating that PAF plays an important role in acetaminophen-induced liver injury and subsequent liver tissue repair, while PAF-AH can increase liver recovery and reduce liver damage [25,26].

The pathological mechanism of hepatitis B is complex, and patients’ symptoms are often complicated by intestinal endotoxemia, the incidence of which can reach 80%–100% in severe hepatitis cases [27]. Lipopolysaccharide (LPS) is the major chemical endotoxin capable of stimulating PAF and PAF-AH secretion from monocytes and macrophages. Kupffer cells, specialized macrophages located in the liver, account for 80%–90% of the total monocytes and macrophages in the body. Kupffer cells are the primary mediators of endotoxin clearance and detoxification in the liver. By injecting bacterial LPS into the rat mesenteric vein, Howard et al. [28] observed a 20-fold increase in the PAF-AH mRNA level in Kupffer cells and a 2-fold increase in serum PAF-AH activity after 24 h. Svetlov et al. [29] reported that cultured primary Kupffer cells could express more PAF-AH mRNA than hepatocytes and had a 20–25-fold higher PAF-AH secretion rate than hepatocytes, indicating that Kupffer cells may be the main source of PAF-AH during liver damage.

We found that the activity of circulating PAF-AH in patients diagnosed with hepatitis B was positively correlated with TBIL, TBA, ALT, AST, TG, and apoB, negatively correlated with ChE, HDL-c, and apoAI, and not correlated with Glu, BMI, Tch, and LDL-c. However, the PAF-AH activity in healthy controls was positively correlated with TBIL, ALT, TG, Tch, LDL-c, and apoB, negatively correlated with HDL-c, and not correlated with Glu, BMI, TBA, AST, and apoAI. The differences in correlations between hepatitis patients and controls, especially in the correlation of PAF-AH with lipids and bile acids, could be explained by a change in the source of PAF-AH during the development of hepatitis. PAF-AH can be classified into intracellular types I and II and the plasma type [3]. Plasma PAF-AH exists in the blood and is predominantly produced by monocytes, macrophages, T lymphocytes, mast cells, and hepatocytes [30-32]. Under normal conditions, the main source of circulating PAF-AH is hematopoietic cells [31], and the main source of PAF-AH in bile juice is hepatocytes [29]. However, during hepatitis bile excretion disorder, the retention of bile components such as bile acid can cause the retention of hepatocyte-secreted PAF-AH [33,34]. This relationship could explain the association between serum PAF-AH activities and the TBIL and TBA levels in hepatitis. Another potential factor is the impact of liver damage on Kupffer cell PAF-AH secretion, leading to an increase in PAF-AH during hepatitis. Circulating PAF-AH mainly exists in complexes with lipoprotein particles [35]. During hepatitis, however, liver damage causes dysfunction in the synthesis of cholesterol and other lipids [36], resulting in the altered correlation between PAF-AH and blood lipids we observed. These findings indicate that serum PAF-AH may be involved in oxidative stress and inflammation of the liver.

We also found that serum PAF-AH activities in patients with various stages of hepatitis B were significantly higher than those in healthy controls, and serum PAF-AH activity was significantly positively correlated with TBIL. The greatest elevation in serum PAF-AH was observed in patients diagnosed with CSHB, suggesting that the PAF-AH activity is involved in pathological liver damage, and that the detection of PAF-AH may serve as a surrogate marker for hepatic inflammatory activity, allowing disease progress and prognosis to be monitored. Similar to our study, the study of Ma et al. found that among CSHB patients, serum PAF activities were significantly higher in the death group than in the control group and were positively correlated with the prognosis of CSHB, and thus can be used as a prognostic factor in CSHB [37]. However, Guerra et al. found that HCV-infected patients showed a significant decrease in PAF-AH activity, and PAF-AH activity recovered only in patients who cleared HCV after antiviral treatment [38]. Most of the circulating HCV is associated with VLDL and LDL, and the circulating HCV lipoprotein complexes may reduce the amount of free LDL available for PAF-AH activity. Since HBV does not circulate in blood bound to LDL, HBV-infected patients did not show any reduction in PAF-AH activity [38,39].

In this study, we show that serum PAF-AH may be used for predicting CSHB and 3-month mortality in HBV-infected patients. We evaluated the predictive power of PAF-AH and the MELD score by ROC curve analysis. The MELD score is a prospectively developed and validated scale for the severity of end-stage liver disease that uses the quantitative, objective values of serum TBIL, serum creatinine, and INR of prothrombin time to predict patient mortality in patients with advanced liver disease [16,40]. Mao et al.’s study reported that the MELD score was related to the prognosis of patients with HBV-related acute-on-chronic liver failure [41]. We found that although the AUC of PAF-AH was lower than the AUC of the MELD score for predicting CSHB, the AUC of PAF-AH combined with MELD score was higher than that of PAF-AH or the MELD score alone. This finding showed that combining PAF-AH with the MELD score further added to predictive power for CSHB. Multivariate logistic regression analysis showed that PAF-AH activity and the MELD score were independent factors predicting CSHB. We also found an association between PAF-AH and mortality in HBV-infected patients. The AUC of PAF-AH was lower than AUC of the MELD score for predicting mortality, and the AUC of PAF-AH combined with the MELD score was similar to that of the MELD score alone. Although this result shows a lower predictive power of PAF-AH than of the MELD score for mortality, PAF-AH activity is an easier and more convenient measurement than the MELD score. However, a potential limitation in our study was that we did not consider the influence of drugs taken before admission that may modulate PAF-AH activity.

In summary, the PAF-AH activities of patients with CSHB were significantly higher than those of patients with AHB, CHB, or LC, and higher PAF-AH activities were associated with a higher prevalence of CSHB. Spearman correlation showed that PAF-AH activity correlated positively with TBIL, TBA, ALT, AST, TG, and apoB and negatively with ChE, HDL-c, and apoAI in patients with hepatitis B. Moreover, serum PAF-AH may be used for predicting CSHB and mortality in patients with hepatitis B, and PAF-AH activity was an independent factor predicting CSHB.

## Abbreviations

PAF-AH: Platelet-activating factor acetylhydrolase; PAF: Platelet-activating factor; HBV: Hepatitis B virus; HCV: Hepatitis C virus; AHB: Acute hepatitis B; CHB: Chronic hepatitis B; CSHB: Chronic severe hepatitis B; LC: Liver cirrhosis; MELD: Model for end-stage liver disease; INR: International normalized ratio; ALT: Alanine aminotransferase; AST: Aspartate aminotransferase; TBIL: Total bilirubin; ChE: Cholinesterase; TG: Triglyceride; Tch: Total cholesterol; LDL-c: Low-density lipoprotein cholesterol; HDL-c: High-density lipoprotein cholesterol; apoAI: apolipoprotein AI; apoB: apolipoprotein B; Glu: Glucose; TBA: Total bile acid; ROC: Receiver operating characteristic; AUC: Area under the curve; BMI: Body mass index; LPS: Lipopolysaccharide.

## Competing interests

The authors declare that they have no competing interests.

## Authors’ contributions

YC designed the experiments. LMF, YZ, and GFF performed the experiments. LMF and YZ wrote the main manuscript text. All authors read and approved the final manuscript.
